# Whole-exome sequencing reveals a novel frameshift and a recurrent nonsense *SPG11* variant causing rare familial amyotrophic lateral sclerosis type 5 in two consanguineous Pakistani families

**DOI:** 10.1007/s11033-026-12618-9

**Published:** 2026-09-01

**Authors:** Riaz Ahmad, Muhammad Naeem, Henry Houlden

**Affiliations:** 1 Medical Genetics Research Laboratory, Department of Biotechnology, Quaid-i-Azam University, Islamabad, 45320 Pakistan; 2 Department of Neuromuscular Disorders, UCL Queen Square Institute of Neurology, Queen Square House, London, WC1N 3BG UK

**Keywords:** Amyotrophic lateral sclerosis, *SPG11*, Spatacsin, Hereditary spastic paraplegias, Exome sequencing

## Abstract

**Background:**

Amyotrophic lateral sclerosis (ALS) is a neurodegenerative disease that affects both upper and lower motor neurons, disturbing communication between the brain and muscles. So far, few reports have been published for the *SPG11*-associated ALS, and this is the first documented case from Pakistan.

**Methods:**

We report a rare subtype of ALS with an autosomal recessive mode of inheritance with juvenile onset before 25 years of age in the five affected individuals from two unrelated families. Whole-exome sequencing was performed to identify disease-causing variants, and selected variants were further prioritized based on predicted pathogenicity and similarity to clinical phenotypes.

**Results:**

Two homozygous variants within the *SPG11* gene were identified as pathogenic according to the ACMG and ClinGen Sequence Variant Interpretation (SVI) Working Group recommendations: a novel truncation (NM_025137.4:c.6738dup, p.Glu2247Ter) variant as validated by Sanger sequencing and a recurrent nonsense (NM_025137.4: c.782C>A, p.Ser261Ter) variant (rs765477482) previously reported in a family affected with autosomal recessive hereditary spastic paraplegia (ARHSP). In silico prediction tools further confirmed their pathogenicity.

**Conclusion:**

Five patients with juvenile-onset ALS born to consanguineous parents were found to have homozygous *SPG11* gene variants. Our findings describe the overlapping phenotypes of*SPG11*-related autosomal recessive juvenile ALS and ARHSP, suggesting that these disorders show a clear overlapping phenotype with common genetic defects. In clinical practice, it is challenging to distinguish between these two disorders. Additional Mendelian cases should be included to clarify further and investigate whether these represent two diverse diseases caused by variants in a single gene.

**Supplementary Information:**

The online version contains supplementary material available at https://doi.org/10.1007/s11033-026-12618-9.

## Introduction

Amyotrophic lateral sclerosis is a neurodegenerative disorder characterized by progressive degeneration of upper and lower motor neurons. Around 90% of ALS cases are sporadic, and the remaining 10% are familial. Genetic factors contribute significantly to both forms of ALS; more than 25 genes have been identified as causative in 60–70% of familial ALS and about 10% of sporadic ALS cases [1]. Many well-known ALS-associated genes exhibit pleiotropic effects and contribute to heterogeneous diseases, thereby connecting ALS to several other conditions, such as spinocerebellar ataxia (SCA), frontotemporal dementia (FTD), inherited myopathies and neuropathies, Parkinson’s disease (PD) and Paget’s disease of bone (PDB) [2]. Furthermore, the discovery of genes like *KIF5A* and *SPG11* that cause inherited spastic paraplegias [3, 4] has now been associated with adult-onset and juvenile forms of ALS, respectively. This overlap further suggests the clinical and genetic link between ALS and hereditary spastic paraplegias.

Amyotrophic lateral sclerosis type 5 (ALS5) is a subtype of ALS having a recessive pattern of inheritance with juvenile onset, before the age of 25 years [5, 6]. Slow rate of progression, early onset, and long disease duration are some of the features that distinguish ALS5 from classical ALS (adult-onset ALS) [7]. Knockout mice showed cognitive and early motor deficits accompanied by neurodegeneration in the motor cortex, cerebellum, hippocampus, and corticospinal tract, underlining the critical role of the affected gene in motor and cognitive function [8]. A study reported 22 patients (7 families) with ALS linked ALS5 to chromosome 15 (15q15-21) and variants in the *SPG11* gene [5, 7]. The *SPG11* gene comprises 40 exons and spans approximately 100 kb, encoding a 2,443-amino-acid protein. Although the precise function of spatacsin in disease etiology remains unclear, it is thought to be involved in axonal maintenance and trafficking, as well as in the recycling of lysosomes from autolysosomes [9, 10]. Over 250 variants have been identified in *SPG11*, most of which are nonsense, frameshift, or splicing variants, likely causing loss of function through truncated proteins in recessive disorders. All these variants are scattered throughout the gene without clear hotspots linked specifically to autosomal recessive hereditary spastic paraplegia, juvenile amyotrophic lateral sclerosis (JALS), or Charcot–Marie–Tooth disease type 2 (CMT2) [11]. The shared etiologies among different disorders raise the question of why individuals bearing *SPG11* variants have diverse clinical manifestations.

Our presented work further explored the spectrum of variants in *SPG11* and the clinical diversity of JALS disorder in two consanguineous families. Our findings underscore the need for comprehensive screening of variants in additional ALS families to improve disease management, genetic counselling, elucidate disease-causing pathogenic mechanisms, and find potential biomarkers. Our findings indicated that the spectrum of *SPG11* variants most associated with ALS is found in populations with high levels of consanguinity, due to an autosomal recessive pattern of inheritance, including some regions in Europe [12]. The same is the case; biallelic variants are extremely high in the Pakistani population, as described previously [13] for inherited neurological disorders diagnosed with whole-exome sequencing. In addition to our findings, pathogenic *SPG11* variants have been documented in neighbouring Iran, with multiple studies identifying homozygous variants in patients from consanguineous families with hereditary spastic paraplegia and overlapping ALS phenotypes [14, 15].

## Materials and methods

### Participants and consent approvals

This study was approved by the Institutional Ethical Committee of Quaid-I-Azam University, Islamabad, Pakistan, following standard Helsinki protocols. Informed consent was obtained from both affected families (family 1 & family 2) before peripheral blood samples and clinical history collection. Pedigrees for both unrelated families were constructed according to the information provided by their senior family members. Photographs of the affected individuals were taken after obtaining written informed consent. Whole blood samples were collected in EDTA tubes for further genetic investigation. A standard protocol was used for DNA extraction as described previously from all the available blood samples of healthy and affected individuals [16].

### Clinical and paraclinical evaluation

All affected subjects underwent clinical examinations conducted by experienced neurologists. Clinical assessment included evaluation of age at onset, disease progression, family history with emphasis on consanguinity, gait, spasticity, dysarthria, hearing impairment, motor skills, and intellectual disability.

Paraclinical evaluations were performed, including magnetic resonance imaging (MRI) of the thoracolumbar spine in subject (IV:2) of family 1. Karyotyping testing was carried out in the affected individual (IV:1) from family 1. Routine laboratory tests, including complete blood count (CBC), thyroid function test and serum calcium level, were performed for the proband of family 2.

### Whole-exome sequencing, runs of homozygosity (ROHs) and Sanger sequencing

For probands of family 1 (IV:1) and family 2 (V:1), whole-exome sequencing was performed through the Agilent SureSelect Human All Exome V6 Kit (Agilent Technologies, Santa Clara, CA, USA). Whole-exome sequencing, alignment, variant calling and annotation were performed by following a standard pipeline [17] using Illumina NovaSeq 6000 sequencing, BWA (Burrows-Wheeler Aligner v0.7.17 (BWA; http://bio-bwa.sourceforge.net/), SAMtools v1.8, Picard v2.18.9, Genome Analysis Toolkit v4.0 (http://www.broadinstitute.org/gatk/) and variant annotation tools including ANNOVAR (http://www.openbioinformatics.org/annovar/) and FILTUS, with variant classification based on ACMG guidelines [18]. The sequencing showed a mean depth of coverage of 142.05X, with approximately 99% of targeted bases covered at ≥ 20X, indicating high-quality and reliable whole-exome sequencing data. To identify regions of homozygosity (ROHs), the AutoMap (https://automap.iob.ch/process) tool with default settings was used for the proband, as described previously [19].

Sanger sequencing was performed to validate and assess the segregation of variants identified through whole-exome sequencing. Before Sanger sequencing, the reference sequence of *SPG11* was obtained from Ensembl (http://www.ensembl.org/). Targeted primers were designed using the Primer3Web (version 4.1.0) tool (https://primer3.ut.ee/) and used to amplify the desired region with the candidate variant. PCR products were purified using a PCR purification kit (GenJet PCR Purification Kit, Thermo Scientific USA, Catalog No: K0701). The PCR products were assessed using a 2% agarose gel, alongside a 1 kb ladder. After subjecting these PCR products to a genetic analyzer (ABI-3730 DNA Analyzer), the generated files were visualised using Sequencher 5.0 software.

### Filtration Criteria

**Fig. 1:**
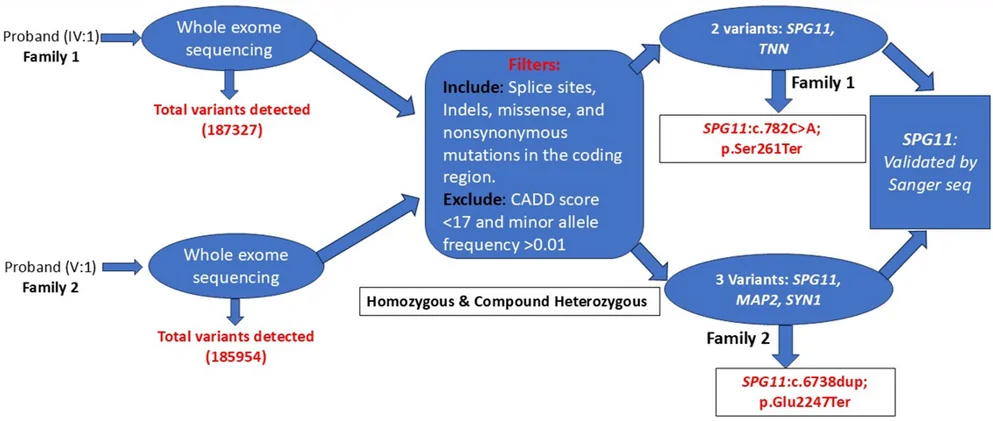
Filtration criteria for variant detection in the probands of two unrelated families

Filtration criteria as outlined in Fig. 1 were applied to both families to identify the pathogenic cause, pattern of inheritance, and clinical correlation with genotypes. During filtration, we focused on coding regions and canonical splice acceptor/donor sites. Rare variants with a minor allele frequency < 0.01% were prioritized. Rare variants, including missense, nonsense, frameshift (insertion & deletion), and splice sites, were further filtered according to the expected inheritance pattern (homozygous or compound heterozygous) and relevant neurological phenotypes.

### Computational Analysis and Evolutionary Conservation

Different in silico predictions were performed as shown in Supplementary Table 1. Pathogenicity of our detected variants was confirmed with MutationTaster (www.mutationtaster.org), CADD (Combined Annotation Dependent Depletion (https://cadd.gs.washington.edu), DANN, BayesDel, GenoCanyon, fitCons, and VarSome (https://varsome.com/). Franklin variant assessment tool (https://franklin.genoox.com/clinical-db/home) was also applied to further classify the variants according to the ACMG guidelines and ClinGen Sequence Variant Interpretation (SVI) Working Group recommendations. To rule out false positives and artifacts, selected variants were visually examined via the Integrative Genomics Viewer (IGV) [20]. NMDEscPredictor (https://nmdprediction.shinyapps.io/nmdescpredictor/) tool was applied to predict nonsense-mediated decay (NMD).

## Results

We report five patients (from two unrelated families) showing a slowly progressive motor neuron disease. Whole-exome sequencing was performed to identify disease-causing variants, and selected variants were further prioritized based on predicted pathogenicity and similarity to clinical phenotypes. In the first step, we filtered (Fig. 1) all the detected variants and shortlisted a few variants (Supplementary Table 2). Even based on our pipeline, ALS5 phenotypes were detected in both probands of our registered families. In both cases, the *SPG11* gene was investigated to carry pathogenic truncating variants as assessed according to the ACMG guidelines and the recommendations of the ClinGen Sequence Variant Interpretation (SVI) Working Group. CADD score for NM_025137.4: c.6738dup, p.Glu2247Ter was 35 and for nonsense (NM_025137.4: c.782 C > A, p.Ser261Ter) variant, 37 was detected. All applied in silico tools are summarized in Supplementary Table 1. Functional status and disease progression were assessed (Table 1) using the Amyotrophic Lateral Sclerosis Functional Rating Scale–Revised (ALSFRS-R), with scores ranging from 29/48 (60.4%) in family 1 and 26/48 (54.2%) in family 2, consistent with progressive juvenile-onset ALS phenotypes [21].

**Table 1:** Comparison of clinical features of the patients ascertained in the present study

Category	Feature	Family 1 – IV:1 (M)	Family 1 – IV:2 (F)	Family 1 – IV:3 (M)	Family 2 – V:1 (M)	Family 2 – V:2
Demographic/Background	Consanguinity	+	+	+	+	+
	Age at onset (years)	17	15	18	17–18	17–18
Disease Course	ALSFRS-R score (0–48)	29/48	29/48	29/48	26/48	26/48
Upper Motor Neuron (UMN) Features	Spastic gait	Mild-moderate	Severe	Moderate	Moderate	Moderate
	Limb spasticity	+ (lower limbs)	+ (Upper and lower limbs)	+ (lower limbs)	+ (lower limbs)	+ (lower limbs)
	Deep tendon reflexes	+ (Increased hyperreflexia)	+(Increased hyperreflexia)	+ (Increased hyperreflexia)	+ (Increased hyperreflexia)	+ (Increased hyperreflexia)
Bulbar UMN Features	Dysarthria/anarthria	Mild-moderate	Severe	Mild-moderate	Severe/anarthria	Mild
	Dysphagia/chewing difficulty	Mild	Severe	Mild–moderate	Severe	Mild
Lower Motor Neuron (LMN) Features	Muscle atrophy	+	+	+	+	+
	Tongue atrophy	+	+	+	+	+
	Fasciculations	+	+	+	+	+
	Muscle weakness	+	+	+	+	+
	Muscle cramps	+	+	+	Occasional	Occasional
	Hypotonia/Flaccidity	+ (Distal upper limbs)	+ (Distal upper limbs)	+ (Distal upper limbs)	+ Upper and lower limbs with marked distal wasting	+ Upper and lower limbs
	Paralysis	Progressing to paralysis	Progressing to paralysis	Progressing to paralysis	Progressing to paralysis	Progressing to paralysis
	EMG evidence of LMN involvement	NA	NA	NA	NA	NA
Sensory System	Sensory loss (pinprick/light touch)	−	−	−	−	−
Cognition/Other Systems	Intellectual disability	−	−	−	+	+
	Ocular/hearing impairment	−	−	−	−	−
Disease Course	Disease progression	Slow	Slow	Slow	Slow	Slow
Neuroimaging	MRI findings	NA	Normal thoracolumbar MRI	NA	NA	NA
	Karyotyping	Normal	NA	NA	NA	NA
Genetics	SPG11 variant	c.782 C > A; p.Ser261Ter (homo)	c.782 C > A; p.Ser261Ter (homo)	c.782 C > A; p.Ser261Ter (homo)	c.6738dup; p.Glu2247Ter (homo)	c.6738dup; p.Glu2247Ter (homo)

**Fig. 2:**
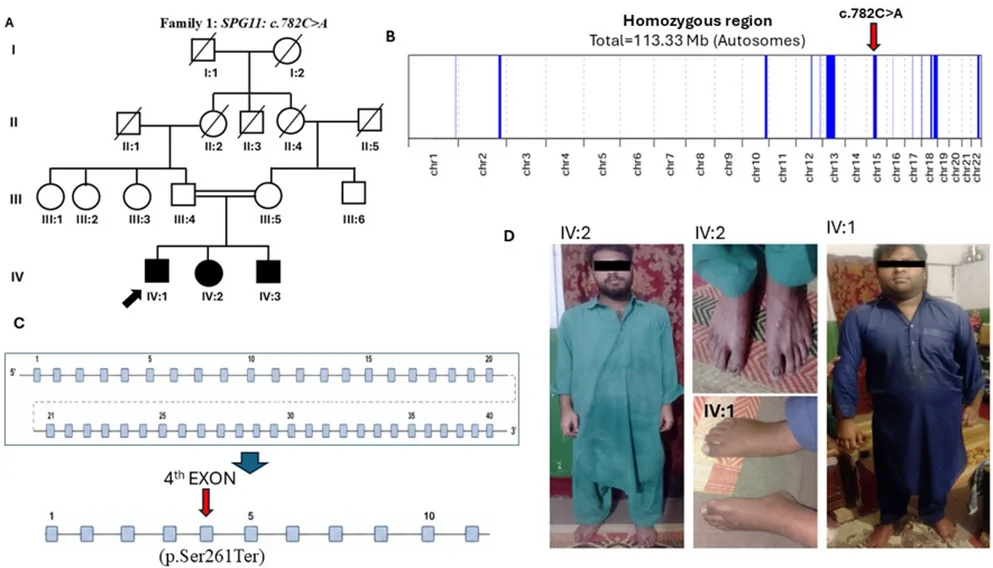
(A) Pedigree of family 1. (B) Homozygosity mapping of the proband (IV:1). (C) *SPG11* gene structure with a total of 40 exons.(D) Photographs of the affected individuals

Family 1 comprised three affected individuals, IV:1 (male), IV:2 (female), and IV:3 (male), born to healthy parents (Fig. 2) in a four-generation pedigree, showing no prior history of neurological disorders in earlier generations or within the extended family loops. The parents of the affected individuals were healthy and showed no symptoms of the disease. Symptoms began at seventeen (17) years of age in the proband (IV:1), fifteen (15) years in IV:2, and eighteen (18) years in IV:3. The proband IV:1 developed clinical features such as mild to moderate spastic gait, increased hyperreflexia, dysarthria, dysphagia, muscle atrophy, and progressing to paralysis, as shown in Table 1. MRI of the thoracolumbar spine was normal as recently examined in the clinical evaluation of subject IV:2. In the same subject, clinical features were more severe (severe difficulty with motor skills such as walking, climbing stairs, grasping objects, speaking, swallowing, and chewing) as compared to the other two affected males. Karyotype testing was also normal for the proband of this family, excluding chromosomal aberrations. The phenotypes of the affected individuals matched with amyotrophic lateral sclerosis 5, juvenile (OMIM: 602099). A recurrent nonsense (NM_025137.4: c.782 C > A, p.Ser261Ter) variant (rs765477482) was detected in this family with an autosomal recessive pattern of inheritance.

**Fig. 3:**
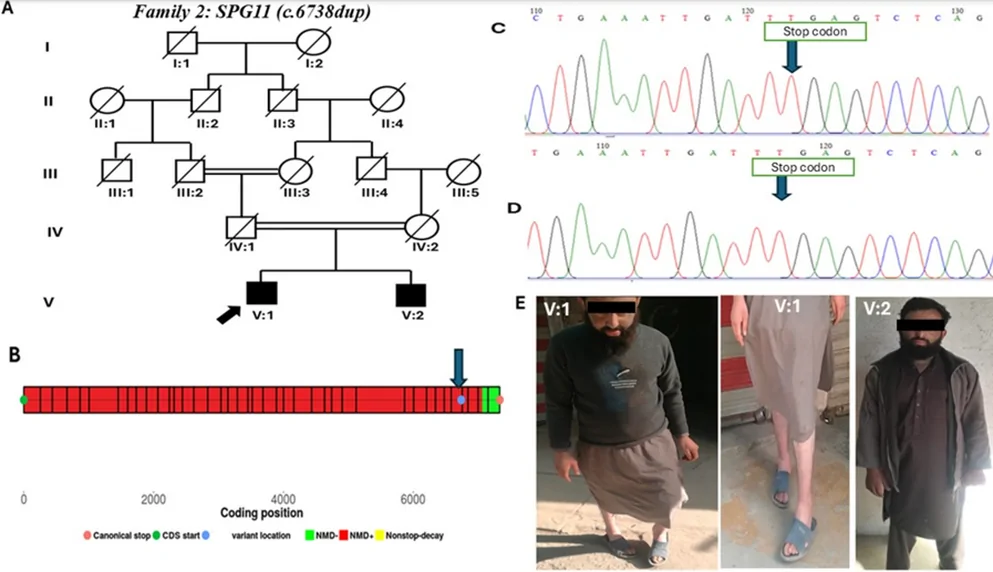
(A) Pedigree of family 2. (B) Nonsense-mediated RNA decay representation. (C & D) Chromatogram of the affected individuals (V:1 & V:2). (E) Phenotypes (subject V:1 & V:2) of family 2

Family 2 comprises two affected individuals (V:1, V:2) born of consanguineous parentage (Fig. 3) with a phenotype of dysarthria, moderate spasticity, increased deep tendon reflexes (hyperreflexia), occasional cramps, and dysphagia with no ocular or hearing impairment. The onset of symptoms was observed at 17 to 18 years of age in both affected subjects. The parents were asymptomatic for any kind of neurological manifestations. Proband (V:1) can’t talk, suggesting severe dysarthria or anarthria, a hallmark of bulbar involvement often observed in ALS progression. Secondly, he is unable to walk properly, having a jerky, unsteady gait consistent with weakness, spasticity, and upper motor neuron signs typical of ALS. Family 2 patients demonstrate lower motor neuron (LMN) involvement, such as muscle atrophy, tongue atrophy, muscle weakness and hypotonia, as summarized in Table 1. Intellectual disability was also seen, but facial dysmorphic features were not observed. Blood-related tests were normal, such as complete blood count (CBC), thyroid function, and calcium level. Based on initial clinical evaluation, this family was suspected to be a case of amyotrophic lateral sclerosis 5, juvenile (602099). A novel truncation (NM_025137.4:c.6738dup, p.Glu2247Ter) variant was identified and further validated by Sanger sequencing. This variant was absent in dbSNPs, 1000 Genomes, and even in our in-house database, including more than 3000 exomes.

**Fig. 4:**
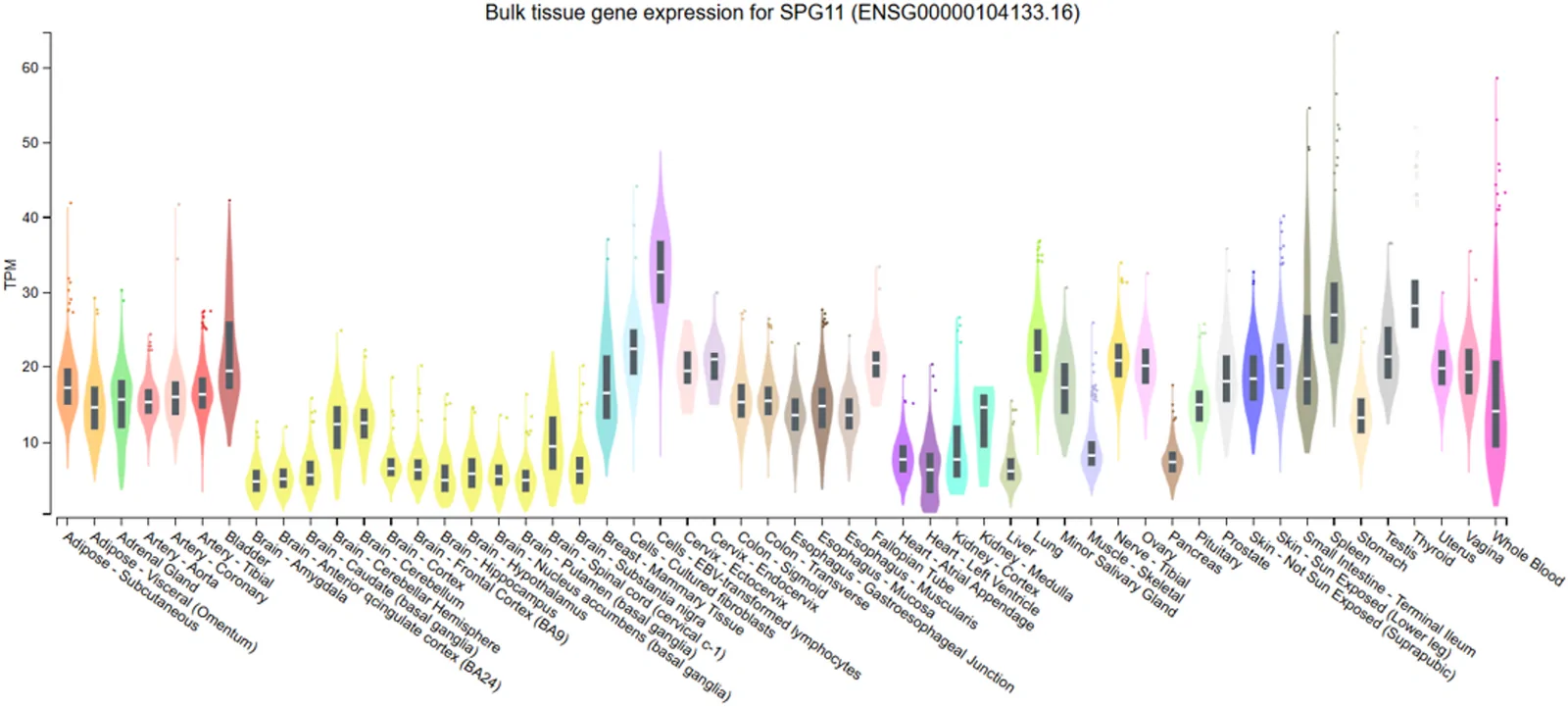
Multiple tissue-specific expression profiles of *SPG11* based on GTEx

*SPG11* is expressed across multiple tissues (Fig. 4), with relatively higher expression in central nervous system (CNS) related tissues, including brain regions relevant to motor neuron function (https://gtexportal.org/home/gene/SPG11).

## Discussion

ALS is an incurable, adult-onset neurodegenerative condition characterized by progressive degeneration of motor neurons, leading to gradual weakness of the limbs, bulbar and respiratory muscles [22]. The ensuing muscle weakness leads to rapid functional decline and reduced ability to perform daily activities [23]. Mortality most commonly occurs due to respiratory failure. No curative therapy exists; current management strategies primarily aim to alleviate symptoms and maintain quality of life [23].

The pathogenic nonsense *SPG11* gene variant c.782 C > A; p.Ser261Ter was identified in Family 1. The same variant was surprisingly reported by Kara et al. in 2016 in a 41-year-old female affected with hereditary spastic paraplegia [24]. She had problems in walking, spasticity, severe bladder problems, ataxia, neuropathy, parkinsonism, and severe optic atrophy. The age of onset was 21 years, and factor VII deficiency was also observed in the patient [24]. Our patients’ phenotypes were completely changed, associated with amyotrophic lateral sclerosis 5, juvenile instead of hereditary spastic paraplegias (HSPs), as shown in Table 1. Both medical and familial history were unremarkable, like the report published by Santos Silva et al. (2023) [25]. Similarly, in our patients, no fasciculations, muscle wasting, or weakness of the upper extremities were evident during the early stage of the disease; however, as the disease progressed, all affected individuals developed generalized muscle weakness, muscle atrophy, and fasciculations, consistent with lower motor neuron involvement.

Overlaps between clinical features of autosomal recessive HSP and juvenile ALS are evident [26–28]. Some features differentiate HSP-11 and ALS5, including bulbar involvement at onset, absence of cognitive impairment and sensory neuropathy, which may aid in distinguishing them [29]. Nevertheless, there is still difficulty with differential diagnosis, as they likely fall within the same neuropathological and clinical spectrum.

In 2010, Orlacchio et al. studied 10 ALS families and identified 12 variants in *SPG11*, 10 of which were either nonsense or frameshift, leading to a loss of function [4]. Later, a compound heterozygous variant in a deletion form was reported with intrafamilial phenotypic heterogeneity [30].

Identified variants in our study (frameshift and nonsense) in *SPG11* are predicted to result in premature termination codons and are therefore most likely to trigger nonsense-mediated mRNA decay (NMD), leading to a loss of function as the primary disease mechanism. Future studies are needed to further elucidate the role of these identified variants in the context of NMD. Our study enrolled two families with five affected subjects presenting with a slow, progressive juvenile motor disease resembling amyotrophic lateral sclerosis type 5. Family 1 harbored the recurrent *SPG11* variant c.782 C > A (p.Ser261Ter), whereas Family 2 carried the novel *SPG11* variant c.6738dup (p.Glu2247Ter). Despite overlaps between ALS and HSP, our findings highlight the diagnostic challenges and phenotypic variability of *SPG11*-associated disorders, reinforcing the key role of genetic testing in classifying clinical presentations.

## Conclusion

Our findings emphasize the necessity for genetic awareness, carrier screening, and use of next-generation sequencing technologies to decrease the burden of inherited neuropathies in consanguineous populations.

## Supplementary Information

Below is the link to the electronic supplementary material.Supplementary material 1 (DOCX 18.0 kb)Supplementary material 2 (XLSX 2260.2 kb)

## Data Availability

All data supporting the findings of this study are available within the paper and its Supplementary Information.
