# Supplementary material for: Whole-exome sequencing reveals a novel frameshift and a recurrent nonsense SPG11 variant causing rare familial amyotrophic lateral sclerosis type 5 in two consanguineous Pakistani families
Source: Mol Biol Rep. 2026 Sep 1;53(1):1504. doi: 10.1007/s11033-026-12618-9 (PMC13534196; doi:10.1007/s11033-026-12618-9)
Supplement: Supplementary file 1 [file 11033_2026_12618_MOESM1_ESM.docx]

Supplementary Table 1. A summarized form of in silico tools used to detect the pathogenicity of the investigated nonsense (NM_025137.4: c.782C>A, p.Ser261Ter) and frameshift (NM_025137.4: c.6738dup, p.Glu2247Ter) variants in *SPG11*.

| **S. No** | **ENGIN** | **Prediction** | |
| --- | --- | --- | --- |
|  |  | NM_025137.4: c.782C>A, p.Ser261Ter **(Family 1)** | NM_025137.4: c.6738dup, p.Glu2247Ter  **(Family 2)** |
| 1 | CADD score | Deleterious (35) | Deleterious (37) |
| 2 | DANN | Deleterious (0.99) | N/A |
| 3 | BayesDel | Deleterious (Strong) (0.66) | N/A |
| 4 | AlphaMissense | N/A | N/A |
| 5 | GenoCanyon | Deleterious (1) | N/A |
| 6 | fitCons | Deleterious (0.74) | N/A |
| 7 | VarSome | Pathogenic | Pathogenic |
| 8 | MutationTaster | Disease-causing (1) | Disease-causing (1) |
| 9 | Human Splicing Finder / SpliceAI | N/A | N/A |
| 11 | ACMG/ClinGen SVI Criteria Applied | (Pathogenic): PVS1(Very Strong):predicted LoF; ClinGen SVI decision tree, PM2_Supporting (absent from population databases),  PP1_Supporting (segregation in affected family members) | (Pathogenic):  PVS1(Very Strong): frameshift leading to premature truncation; LoF mechanism), PM2_Supporting (absent from population databases), PP1_Supporting (segregation in affected family members) |
